# Economic valuation of health benefits from using geologic data to communicate radon risk potential

**DOI:** 10.1186/s12940-020-00589-8

**Published:** 2020-03-20

**Authors:** Scott J. Chiavacci, Carl D. Shapiro, Emily J. Pindilli, Clyde F. Casey, Mary Kay Rayens, Amanda T. Wiggins, William M. Andrews, Ellen J. Hahn

**Affiliations:** 1 Science and Decisions Center, United States Geological Survey, 12201 Sunrise Valley Dr, Reston, VA 20191 USA; 2 University of Kentucky College of Nursing, BREATHE, Lexington, KY 40536 USA; 3 Kentucky Geological Survey, University of Kentucky, Lexington, KY 40506 USA

**Keywords:** Economics, Geologic map data, Kentucky, Lung cancer, Radon, Smoking

## Abstract

**Background:**

Radon exposure is the second leading cause of lung cancer worldwide and represents a major health concern within and outside the United States. Mitigating exposure to radon is especially critical in places with high rates of tobacco smoking (e.g., Kentucky, USA), as radon-induced lung cancer is markedly greater among people exposed to tobacco smoke. Despite homes being a common source of radon exposure, convincing homeowners to test and mitigate for radon remains a challenge. A new communication strategy to increase radon testing among Kentucky homeowners utilizes fine-scale geologic map data to create detailed radon risk potential maps. We assessed the health benefits of this strategy via avoided lung cancer and associated premature mortality and quantified the economic value of these benefits to indicate the potential utility of using geologic map data in radon communication strategies.

**Methods:**

We estimated the change in radon testing among all 120 counties in Kentucky following a new communication strategy reliant on geologic maps. We approximated the resultant potential change in radon mitigation rates and subsequent expected lung cancer cases and mortality avoided among smokers and non-smokers exposed to 4 pCi/L of radon in the home. We then applied the value of a statistical life to derive the economic value of the expected avoided mortality.

**Results:**

The new communication strategy is estimated to help 75 Kentucky residents in 1 year avoid exposure to harmful radon levels via increased testing and mitigation rates. This equated to the potential avoidance of approximately one premature death due to lung cancer, with a net present value of $3.4 to $8.5 million (2016 USD).

**Conclusions:**

Our analysis illustrates the potential economic value of health benefits associated with geologic map data used as part of a communication strategy conveying radon risk to the public. Geologic map data are freely available in varying resolutions throughout the United States, suggesting Kentucky’s radon communication strategy using geologic maps can be employed in other states to educate the public about radon. As this is only a single application, in a single state, the economic and health benefits of geologic map data in educating the public about radon are likely to exceed our estimates.

## Background

Exposure to radon is the second leading cause of lung cancer [1] and is responsible for 15,000 to 22,000 lung cancer deaths per year in the United States [2]. Further, most radon-induced lung cancer cases occur in persons also exposed to tobacco smoke [3]. Recent studies also suggest an association between radon exposure and deaths from skin cancer and breast cancer [4, 5]. Considering about 7% of homes in the United States contain harmful levels of radon [6], this radioactive gas poses a serious threat to millions of people.

Morbidity and mortality resulting from radon exposure carry substantial costs to both individuals and society. For example, just the first year of treatment for radon-caused lung cancer in Utah was estimated to cost an average of between $2.7 and $3.6 million (based on data from 2002 to 2011), excluding costs from lost productivity and premature death [7]. Thus, finding ways to reduce or eliminate the public’s exposure to radon can have measurable health and economic benefits (e.g., [8–11]).

Though the health effects resulting from radon exposure in the home are largely preventable through testing and mitigation, addressing radon exposure is challenging for several reasons. For example, although the common location where most people are exposed to harmful radon levels is in the home [12, 13], many homeowners show indifference to radon risk, do not understand the health risks posed by radon exposure or their likelihood of being exposed to it, or they may not know how to test and mitigate for it (e.g., [14, 15]). In some cases, even when residents in an area have heard of radon, they may possess little understanding of the degree to which it poses health risks where they live (e.g., [16]). The challenge of minimizing radon exposure is exacerbated by it being odorless, tasteless, and invisible [17], properties that make detecting radon impossible without testing. Although testing for radon is easy and affordable ($15 per short-term charcoal-based test kit; 2019 USD), radon mitigation and associated repairs can be costly (e.g., $800 to $2000 per home [2019 USD] depending on the size of the home and the foundation [18];). These potential costs may discourage some residents from testing their homes [19], especially in the absence of information about the potential for homes in certain locations to contain harmful levels of radon. With additional information, residents might decide that testing and mitigation, if needed, are worth the cost. Lastly, laws requiring radon testing (e.g., as part of a real estate transaction) vary by state in the United States. All of these circumstances contribute to a continuing avoidable health risk.

Kentucky residents are at particularly high risk of developing lung cancer from radon exposure for several reasons. First, the state’s geologic composition contains rocks with locally high levels of uranium, an element that releases radon as it breaks down in rocks and soil [17]. Specifically, it is estimated that 42% of homes in Kentucky have radon levels at or above the United Sates Environmental Protection Agency (USEPA) action level of 4.0 pCi/L (Radon Policy Division: Kentucky Geologic Radon Potential Map Project, Kentucky Residential Radon Registry, unpublished), likely because Kentucky’s average radon levels are among the highest in the United States [20]. Secondly, tobacco smokers are at increased risk of developing lung cancer due to radon exposure than never-smokers [3]. Because 24.5% of Kentucky’s population smokes [21] and only about one-third of the state’s population is protected by strong smoke-free workplaces and enclosed public places laws [22], many residents face an increased likelihood of developing lung cancer due to the synergistic effects of tobacco smoke and radon exposure.

**Fig. 1:**
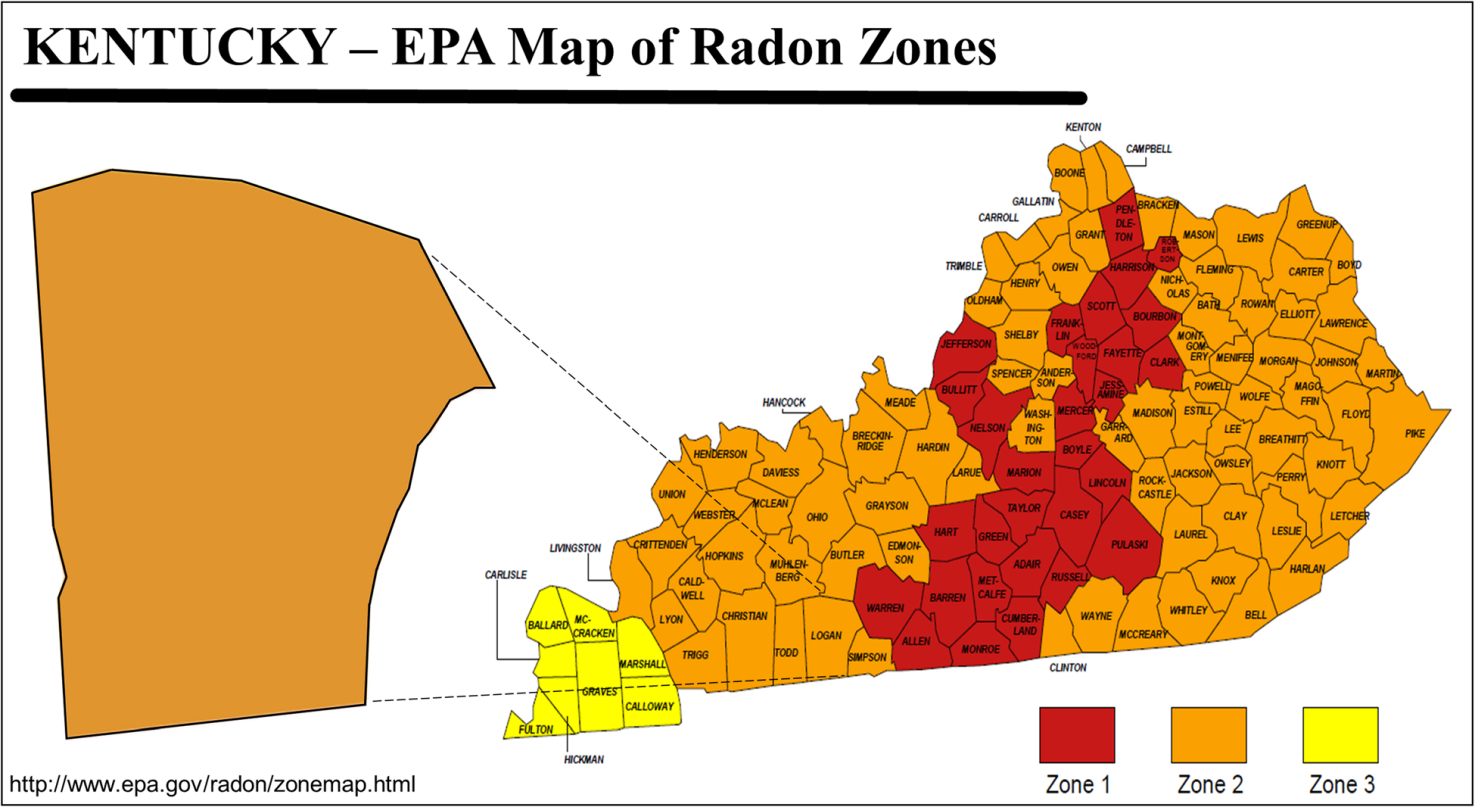
Example of radon zone map produced by United States Environmental Protection Agency and previously used by the state of Kentucky, USA to illustrate radon risk potential. Zone 1 represents counties with predicted average radon screening levels > 4 pCi/L, Zone 2 represents counties with predicted average radon screening levels of 2 to 4 pCi/L, and Zone 3 represents counties with predicted average radon screening levels < 2 pCi/L. Image is a United States government work not subject to copyright [26]. Image content has been slightly reorganized and enlarged to improve interpretability

**Fig. 2:**
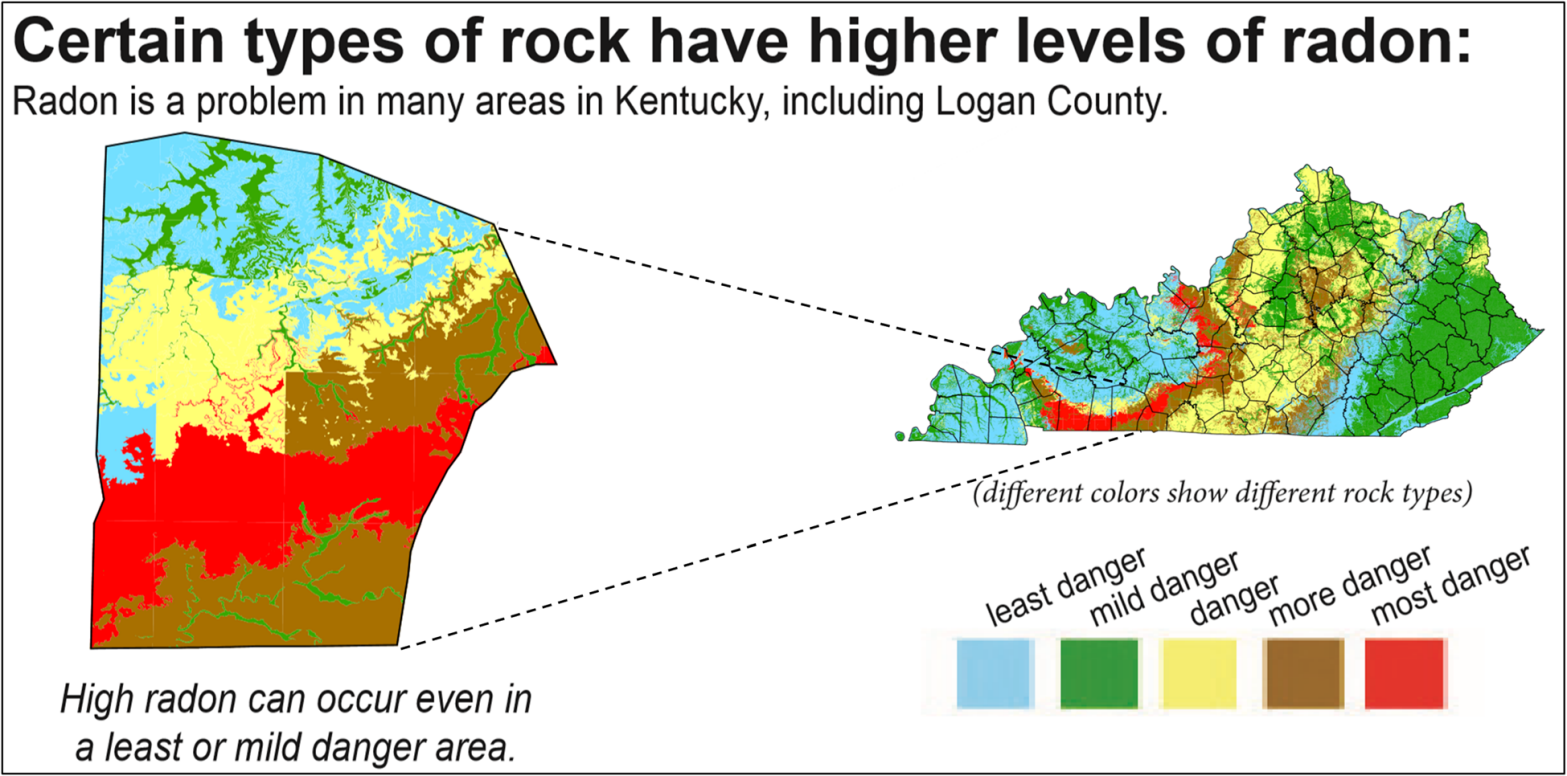
Example of newly developed map showing radon danger levels based on home testing and geologic data in the state of Kentucky, USA to illustrate radon risk potential. Radon levels by rock type (pCi/L) ranged from 0.0–2.7 (blue), 2.71–4.0 (green), 4.01–8.0 (yellow), 8.01–16.00 (brown), 16.01–25.30 (red). Image produced and used with permission from the Kentucky Geological Survey [24, 25]

In response to this health risk, public health practitioners and researchers from the University of Kentucky College of Nursing and geologists from the Kentucky Geological Survey engaged in a collaborative effort to inform residents of Kentucky, USA, about radon risk potential where they live and motivate them to test for and mitigate radon in their homes [23]. The effort included the development of highly detailed radon risk potential maps that combined detailed geologic map data [24, 25] and observed radon values from over 60,000 home test kits. In addition, infographics were developed to enhance the visualization of radon risk potential in the state as part of a broader communication strategy. Prior to the development of these detailed geologic maps, USEPA maps were used that identified each county as belonging to 1 of 3 possible radon zones (Fig. 1 [26];). The new detailed geologic maps convey 5 radon potential danger levels throughout the state at a finer resolution than previously-used maps [Fig. 2 (Radon Policy Division: Kentucky Geologic Radon Potential Map Project, Kentucky Residential Radon Registry, unpublished);].

Our objective was to estimate the potential public health outcomes and associated economic benefits of using these highly detailed geologic maps as part of a communication strategy to reduce radon risk potential. It is important to note that we do not attempt to disaggregate the benefits of the data, detailed maps, or additional communication. Rather this analysis should be interpreted as the value associated with the information package and additional communication efforts. To achieve this, we first calculated the change in radon testing rate before and after the dissemination of these more detailed maps. We then used this change in testing rate to forecast the potential impact of more detailed geologic maps on radon mitigation rates, lung cancer incidence, the estimated number of premature deaths avoided, and the economic value of lung cancer avoidance.

## Methods

### Study population and outreach

The Commonwealth of Kentucky, with an estimated population of over 4.4 million people living in 120 counties [27] is in the eastern United States. The geologic radon risk potential maps and user-friendly educational infographics were initially disseminated as a pilot outreach project in 15 counties located in central, western, eastern, and northern Kentucky based on high radon risk potential, existence of a county radon program, and low rates of radon testing (we do not list county names here because of human subject confidentiality guidelines). The goal of the initial outreach project was to gauge interest and usability of these geologic map-based infographics. Outreach was comprised of four in-person (July to September 2016) and four on-line (January and February 2017) workshops with public health professionals. In addition, participants were asked to distribute the maps and other educational materials during their regular outreach activities in the 15 counties. Following the initial outreach project, the maps and educational infographics were posted to the BREATHE website for all 120 Kentucky counties (see http://www.uky.edu/breathe/radon/radon-data-county) by March 2017 and disseminated via press release and social media. During the outreach and dissemination period, there were no other changes to the state or local radon programs.

### Economic benefits estimation

We used a 5-step process to estimate the economic benefits of the new radon communication strategy that incorporated geologic map data. Hereafter, our use of the term ‘risk’ relates to the risk of residents being exposed to radon and its detrimental health impacts (i.e., a public health risk).

In the first step, we determined the number of additional homes tested for radon following use of the detailed geologic maps, as expressed in eq. (1):

1 $$ \boldsymbol{K}=\left(\frac{{\boldsymbol{R}}_{\boldsymbol{t}}}{\mathbf{10,000}}-\frac{{\boldsymbol{R}}_{\boldsymbol{t}-\mathbf{1}}}{\mathbf{10,000}}\right)\times {\boldsymbol{H}}_{\boldsymbol{KY}}\times \mathbf{1}\mathbf{2}\ \boldsymbol{months} $$

where *K* is the change in the number of Kentucky homes tested for radon in a single year following incorporation of geologic maps in the communication strategy, *R*_*t*_*/10,000* is the rate of radon testing per 10,000 homes per month following incorporation of geologic map-based infographics, *R*_*t-1*_*/10,000* is the rate of testing per 10,000 homes per month prior to use of the geologic map-based infographics, and *H*_*KY*_ is the number of homes in Kentucky.

To determine testing rates before and after implementation of the new communication strategy, we used de-identified (i.e., not attributable to any individual person) radon testing data from all 120 Kentucky counties, which were acquired from two major radon testing companies serving the state from 1985 to 2016. These data were analyzed by the BREATHE research team at the University of Kentucky College of Nursing (University of Kentucky College of Nursing: Freedom from Radon Exposure and Smoking in the Home (FRESH) Database, unpublished). Testing rates prior to use of geologic map-based infographics were from September 2014 to June 2015, whereas testing rates following the outreach activities to disseminate the infographics were from September 2016 to June 2017; in both cases, testing rates were derived from a complete census of test results (i.e., they do not represent a sample of testing rates) (University of Kentucky College of Nursing: Freedom from Radon Exposure and Smoking in the Home (FRESH) Database, unpublished). Restricting our analysis to a 10-month period before and after geologic maps were used in the radon communication strategy is a potential limitation of this study (see Discussion). However, we hypothesized that the limited timeframe minimizes potential temporal variation in factors that are cyclical over long time periods (e.g., housing sales and associated radon testing). We used United States Census Bureau data to acquire the number of homes in Kentucky during 2012–2016 [28]. We multiplied the difference in testing rates by 12 months to convert the value from a monthly to a yearly value.

In the second step, we estimated the number of potential additional homes tested for radon owing to the new communication strategy that are expected to contain radon levels ≥4 pCi/L and are subsequently mitigated to reduce radon concentrations, as expressed in eq. 2:

2 $$ \boldsymbol{M}=\boldsymbol{K}\times \%{\boldsymbol{H}}_{\mathbf{\ge}\mathbf{4}\frac{\mathbf{pCi}}{\mathbf{L}}}\times \%{\boldsymbol{H}}_{\boldsymbol{mitigated}} $$

where *M* is the number of additional homes mitigated to reduce radon concentrations, *K* is the change in the number of Kentucky homes tested for radon in a single year following implementation of the communication strategy using geologic map data (value generated in eq. 1), %*H*_≥4 pCi/L_ is the percentage of tested Kentucky homes estimated to contain radon levels at or above the USEPA action level for harmful radon concentrations (i.e., ≥ 4.0 pCi/L), and %*H*_*mitigated*_ is the percentage of Kentucky homes with radon concentrations ≥4.0 pCi/L) expected to be mitigated to lower radon concentrations.

Approximately 42% of Kentucky homes tested for radon contain concentrations at or above the USEPA action level of 4.0 pCi/L (Radon Policy Division: Kentucky Geologic Radon Potential Map Project, Kentucky Residential Radon Registry, unpublished). We estimated, based on the results of a randomized controlled trial in the state [29] that the percentage of these tested homes containing radon concentrations ≥4.0 pCi/L that are mitigated ranges from 17 to 22%; we used an average mitigation rate of 20% for our calculations. For this analysis, we assumed that home mitigation actions were successful in reducing radon-induced lung cancer risk to zero, all else being equal.

In the third step, we calculated the risk of developing radon-induced lung cancer among Kentucky residents, as expressed in eq. 3:

3 $$ \boldsymbol{R}=\left(\%{\boldsymbol{P}}_{\boldsymbol{smokers}}\times {\boldsymbol{LC}}_{\boldsymbol{smokers}}\right)+\left(\%{\boldsymbol{P}}_{\boldsymbol{non}-\boldsymbol{smokers}}\times {\boldsymbol{LC}}_{\boldsymbol{non}-\boldsymbol{smokers}}\right) $$

where *R* is the weighted risk of Kentucky residents developing lung cancer due to radon exposure, %*P*_*smokers*_ is the percentage of Kentucky’s population that smokes tobacco, *LC*_*smokers*_ is the likelihood of tobacco smokers developing lung cancer when also exposed to radon concentrations of 4.0 pCi/L over their lifetime, %*P*_*non* − *smokers*_ is the percentage of Kentucky’s population that does not smoke tobacco, and *LC*_*non* − *smokers*_ likelihood of non-smokers developing lung cancer when exposed to radon concentrations of 4.0 pCi/L over their lifetime. We weighted lung cancer risk by the percentage of Kentucky’s population that do and do not smoke tobacco to account for differences in lung cancer rates between smokers and non-smokers exposed to the same concentrations of radon [3, 6]. This weighting allowed us to use a single value for estimating the risk a given Kentucky resident would develop lung cancer when exposed to a radon concentration of 4.0 pCi/L. As of 2016, the percentage of Kentucky’s population that smoked tobacco was 24.5% [21]. It is estimated that 6.2% (62 out of 1000) of smokers exposed to 4.0 pCi/L of radon over their lifetime could develop lung cancer [3, 6]. In contrast, 75.5% of Kentucky’s population did not smoke tobacco as of 2016 [21] and it is estimated that only 0.7% (7 out of 1000) of non-smokers exposed to 4.0 pCi/L of radon over their lifetime could develop lung cancer [3, 6]. Importantly, our estimates of potential lung cancer development due to radon exposure are conservative, as 4.0 pCi/L is the level at which the USEPA recommends mitigation [6]; there is no known safe concentration of radon, as lower concentrations may also carry health risks [1]. Further, the likelihood of developing lung cancer increases with greater radon concentrations, so levels > 4.0 pCi/L are expected to increase the likelihood of lung cancer development in smokers and non-smokers [3, 6], though this relationship may not be linear.

In the fourth step, we estimated the number of Kentucky residents potentially avoiding premature mortality due to radon-induced lung cancer as a result of the communication strategy using geologic map data, as expressed in eq. 4:

4 $$ \boldsymbol{A}=\left(\boldsymbol{M}\times {\boldsymbol{P}}_{\boldsymbol{home}}\right)\times \boldsymbol{R}\times \boldsymbol{Z} $$

where *A* is the number of Kentucky residents avoiding mortality due to radon-induced lung cancer, *M* is the number of additional homes mitigated to reduce radon concentrations (value generated in eq. 2), *P*_*home*_ is the average number of Kentucky residents per home, *R* is the risk of Kentucky residents developing radon-induced lung cancer (value generated in eq. 3), and Z is the 2-year mortality rate post lung-cancer diagnosis.

We used United States Census Bureau data to acquire the average number of residents per home in Kentucky during 2012–2016 [28]. We used this value to convert the number of additional homes mitigated to the number of persons expected to be affected by the change in testing and mitigation rates. Once diagnosed with lung cancer, approximately 70% of victims die within 2 years [30]. Although mortality rates increase beyond 2 years, we use only the 2-year rate to be conservative in our estimates [30]. Thus, we assumed that among the residents avoiding premature mortality, 70% would have otherwise died within 2 years of diagnosis if not for the potential increase in testing and mitigation resulting from the new radon communication strategy.

In the fifth step, we estimated the potential economic value associated with the geologic map-based infographics by monetizing avoided premature mortality associated with lung cancer resulting from additional radon testing and mitigation. We monetized avoided mortality via the Value of a Statistical Life (VSL) [31], which approximates a person’s willingness to pay (WTP) to avoid premature death. The USEPA estimated the 2015 VSL as $8.7 million USD [32]. Importantly, we did not incorporate morbidity in our analysis. Thus, the economic values we estimate should be considered a lower bound. To account for the fact that the benefits of the new communication strategy will not be realized until premature deaths from radon-induced lung cancer cases are avoided in the future, we incorporated expected time frames from initial radon exposure to lung cancer development. On average, it takes 5 to 15 years of exposure to harmful radon levels to cause lung cancer [3, 12]. To incorporate this variability, we used 5-, 10-, and 15-year exposure periods as our exposure-to-diagnosis windows. To each of these we added 2 years to account for the 2-year post-diagnosis expectation of mortality. We therefore consider avoided premature mortality at 7-, 12-, and 17-years post-radon exposure (2016 is the base year so point estimates are for 2023, 2028, and 2033).

Given that the VSL is expected to increase as real income increases (i.e., as people’s income increases they are willing to pay more to avoid premature mortality), we used income-based WTP adjustment factors from the USEPA [32] to project the VSL for 2023, 2028, and 2033 (these factors are projected through 2026 in the current [July 2018] version of BeneMAP [32]). To determine VSL beyond 2026, we assumed the income-based WTP adjustment factor from 2026 remains constant for years 2027 through 2033 (i.e., income growth is constant). We then converted all the values to the year of our analysis (2016) using Bureau of Labor Statistics inflation factors. Finally, we calculated the net present value (NPV) of avoided premature mortality using 3 and 7% discount rates; this provides a normalized value in current dollar terms [33] by considering peoples’ time-value of money (i.e., one would prefer a benefit today over a year from now). We did not include the cost of testing in our calculations because Kentucky residents have access to free test kits. We also excluded the cost of mitigation in our calculations because our analysis was designed to address only the benefits of geologic map-based infographics communication strategy. We address potential mitigation costs in our Discussion.

## Results

### Step 1

The rate of radon testing in Kentucky immediately prior to the new communication strategy using geologic map data was 1.714 homes per 10,000 homes per month. The testing rate following employment of the new communication strategy was 1.888 per 10,000 homes per month. Multiplying the difference in these rates by the number of households in Kentucky (1,718,217) and converting the estimate from months to a year revealed that in 1 year, the communication strategy incorporating geologic map data potentially resulted in 359 additional homes being tested for radon.

### Step 2

We estimated that 151 (42%) of the 359 additional homes tested for radon are expected to have radon concentrations ≥4 pCi/L; this is likely a conservative estimate if higher rates of testing occur in high risk areas. Assuming 20% of these 151 homes with dangerous radon concentrations engaged in mitigation measures, we expected 30 homes would have reduced radon concentrations to near 0 pCi/L as a result of the new communication strategy.

### Step 3

In 2016, the risk of smokers in Kentucky developing lung cancer when exposed to radon concentrations of 4 pCi/L over their lifetimes was 0.015. The risk of non-smokers developing lung cancer when exposed to radon concentrations of 4 pCi/L over their lifetimes was 0.005. Thus, the weighted risk of a Kentucky resident developing lung cancer was 0.02.

### Step 4

Given an average of 2.49 residents per home in Kentucky, the 30 homes expected to reduce radon concentrations through mitigation measures would result in 75 residents avoiding exposure to dangerous radon levels in a single year due to the new communication strategy. Assuming these 75 residents would have otherwise been exposed to a radon concentration of 4 pCi/L over their lifetimes that carried a weighted lung cancer risk of 0.02, approximately 2 residents could have developed lung cancer. With a 70% mortality rate in the first 2 years post lung cancer diagnosis, approximately 1 of these 2 people could be expected to suffer premature mortality due to lung cancer. Thus, by incorporating geologic map data into a radon communication strategy, Kentucky potentially helped 1 resident avoid premature mortality due to radon-induced lung cancer.

### Step 5

Using the VSL for 2023, 2028, and 2033, we estimated the single Kentucky resident’s future expected WTP to avoid premature death would be $10.4, $10.6, and $10.8 million USD, respectively. Calculating the NPV in 2016 USD, the benefits associated with the new radon communication strategy was estimated at $3.4, $4.7, and $6.5 million USD assuming a 7% discount rate and $6.5, $7.4, and $8.5 million USD assuming a 3% discount rate (NPVs for each discount rate represent values for life expectancies of 17, 12, or 7 years post-lung cancer diagnosis, respectively).

## Discussion

The potential economic and health benefits of the new communication strategy that incorporates geologic map data to convey radon risk potential in Kentucky appear considerable. We propose that the use of detailed geologic map data as part of this strategy potentially increased rates of radon testing because such data more effectively communicated radon risk potential to residents using finer spatial scales than previous maps showing risk information at the course county-level scale. The more detailed geologic maps better illustrate fine-scale variation in radon risk potential, which may better incentivize residents to test for radon. Although the resolution of geologic data in Kentucky is higher than in many other areas of the United States, geologic data are free, publicly-accessible, and available in varying resolutions throughout the country [34]. Importantly, the incorporation of geologic maps in a strategy to communicate radon risk potential does not require the most detailed geologic data – such maps can be developed with the best available scale of geologic mapping. Thus, other states and countries engaged in campaigns to promote radon testing may decrease lung cancer incidence and generate economic benefits by using geologic data to provide more detailed information to residents on the radon risk potential near their homes.

Radon-induced lung cancer is a major health concern within and outside the United States [1, 17] and identifying ways to increase radon testing is an ongoing challenge [14, 15, 19, 35, 36]. However, with proper mitigation, exposure to high levels of radon is avoidable. Based on our estimates, better communicating the relative risk of radon exposure across the landscape to increase radon testing rates has the potential to save many lives and tens of millions of dollars each year in the United States by reducing premature mortality caused by radon-induced lung cancer. Using geologic data to illustrate radon risk potential represents a promising strategy for urging more homeowners to test for radon. Indeed, persons that participated in the outreach programs designed to gauge interest and usability of the geologic map-based infographics expressed the potential of these infographics to increase radon testing rates in Kentucky. A similar communication strategy, coupled with easier access to low-cost or free radon tests, would provide an even stronger incentive for reducing the number of residents exposed to high radon levels [19]. For example, integrating the dissemination of the geologic map-based infographics with free test kits at pharmacies, primary care clinics, and tobacco treatment services could better reach high risk populations [19].

We acknowledge several limitations to our analysis and offer directions for future research into the potential incorporation of geologic map data when conveying radon risk potential. First, factors other than, or in addition to, the geologic map-based infographics may have helped boost radon testing rates among Kentucky residents. For example, other public education sources including social media and radon test kit giveaways over the pre- and post-map period and during National Radon Action Month (January of each year) may have impacted radon testing rates. However, there were no changes to state or local radon programs before, during, or after disseminating the geologic map-based infographics. Second, data on testing rates after release of the geologic map-based infographics were collected over a relatively short time period and the results have not been proven to be statistically different. Continuing to monitor radon testing rates into the future will help discern if the increase in testing following the implementation of the new communication strategy using geologic maps is a short or long-term trend. Longer monitoring of testing rates will also facilitate an examination of any sustained economic benefits of the new communication strategy, as our study calculated only the NPV for a single year, not a recurring, annual value. Fourth, data on actual mitigation is not available in Kentucky; we approximated mitigation rates using estimates from a large randomized controlled trial conducted in the state [29]. Further health and economic studies are needed to more accurately approximate the rates as well as the cost of actual radon mitigation. The average cost to mitigate a home to reduce radon concentrations is $1314 USD (range: $751 - $1878 USD; values adjusted from 2019 to 2016 USD [18]). Although this cost may be large for individual homeowners, it would have had little impact on estimated economic benefits of the new communication strategy employed in Kentucky, given that only 30 homes were expected to undergo mitigation at a total cost of $39,599 USD. However, the costs of testing and mitigation may be useful to consider when determining whether to deploy a similar communication strategy elsewhere. Fifth, research is needed to assess possible differences in testing and mitigation rates (e.g., behavioral responses) among smokers and non-smokers, as has been found in other studies (e.g., [9]). Lastly, we did not consider exposure to secondhand smoke as a cause of lung cancer in this analysis; given the available data, we were able to estimate lung cancer rates among only smokers and non-smokers. There is evidence, however, suggesting non-smokers exposed to secondhand smoke may be at greater risk of developing lung cancer due to radon exposure than persons not exposed to secondhand smoke [37]. Considering Kentucky has one of the highest rates of tobacco smoking in the country and generally weak smoke-free protections [22], a large segment of the population presumably is exposed to secondhand smoke and at an elevated risk for lung cancer due to radon exposure. Given these possibilities and our use of conservative values in our calculations, our estimate of the value of geologic map data may be an underestimate of their true value in terms of lives saved, assuming they may also help reduce lung cancer incidence among residents exposed to secondhand smoke.

## Conclusions

Our study demonstrates the potential economic and public health benefits derived from using detailed geologic data as a communication strategy in the promotion of radon testing to reduce lung cancer incidence. Given the low cost and potential high return of using such data to convey geographic variation in radon concentrations, other states and countries should consider this approach as part of their communication strategy for educating residents about radon risk potential and convincing them to test radon levels in their homes.

## Data Availability

Most of the data used in this paper are publicly available. However, the radon testing dataset generated during the current study are not publicly available due to a confidentiality agreement with the radon testing labs but are available from the corresponding author on reasonable request.

## Abbreviations

BREATHE: Bridging Research Efforts and Advocacy Toward Healthy Environments
NPV: Net present value
pCi/L: Picocuries per liter
USA: United States of America
USD: United States dollars
USEPA: United States Environmental Protection Agency
VSL: Value of a statistical life
